# Significance of myocardial injury on in-hospital clinical outcomes of in-hospital and COVID-19 patients

**DOI:** 10.34172/jcvtr.2023.31614

**Published:** 2023-06-29

**Authors:** Pooja Vyas, Ashish Mishra, Kunal Parwani, Iva Patel, Dhara Dhokia, Trishul Amin, Prarthi Shah, Tanmay Boob, Rujuta Parikh, Radhakishan Dake, Khamir Banker

**Affiliations:** U. N. Mehta Institute of Cardiology and Research Centre (UNMICRC), Civil Hospital Campus, Asarwa, Gujarat, India

**Keywords:** COVID-19, Acute Myocardial Injury, Mortality, High-Sensitivity Troponin I

## Abstract

**Introduction:**

Acute Myocardial injury defined by increased troponin I level is associated with poor in-hospital outcomes and cardiovascular complications in patients with COVID-19. The current study was designed to determine the implications and clinical outcome of myocardial injury in COVID-19.

**Methods:**

This retrospective study included hospitalized COVID-19 patients. Myocardial injury was defined by high sensitivity Troponin I (hs-TNI)≥26ng/l. Cardiac biomarkers, inflammatory markers and clinical data were systemically collected and analyzed. Hazard ratio for in-hospital mortality and logistic regression for predictors of acute myocardial injury were analyzed.

**Results:**

Of the 1821 total patients with COVID-19, 293(16.09%) patients died and 1528 (83.91%) patients survived. Patients who died had significantly higher association with presence of cardiovascular risk factors, severe CTSS ( CT severity score ) and myocardial injury as compared to survived group. 628 (34.5%) patients had evidence of myocardial injury and they had statistically significant association with cardiovascular risk factors, in-hospital mortality, procalcitonin; higher hospital, and ICCU stay. We found significant hazard ratio of diabetes (HR=2.66, (CI:1.65-4.29)), Severe CT score (HR=2.81, (CI:1.74-4.52)), hs-TNI≥26 ng/l (HR=4.68, (CI:3.81-5.76)) for mortality. Severe CTSS score (OR=1.95, CI: 1.18-3.23, *P*=0.01) and prior CVD history (OR=1.65, CI:1.00-2.73, *P*=0.05) were found significant predictors of myocardial injury in regression analysis.

**Conclusion:**

Almost one third of hospitalized patients had evidence of acute myocardial injury during hospitalization. Acute myocardial injury is associated with higher hospital and ICCU stay, mortality, higher in-hospital infection which indicates more severe disease and the poor in-hospital outcomes.

## Introduction

Globally we have 530 896 347 confirmed cases of COVID 19, including 6,301,020 deaths reported to WHO till date 6^th^ June 2022.^1^ Patients with coronavirus disease-19 (COVID-19) caused by severe acute respiratory syndrome-coronavirus-2 (SARS-CoV-2) are also found to have extra pulmonary impact of the disease. SARS-COV-2 enters into cardiac myocytes, vascular tissues and circulating cells though ACE2 (angiotensin converting enzyme-2) receptor present in host cells for the viral spike protein. SARS-COV-2 may directly infect cardiac cells as well can indirectly damage cardiac tissues due to hypoxia, dysfunctional immune response, microvascular injury and microvascular thrombosis.^2^

Prognosis and clinical outcomes are poor in patients with underlying heart disease and risk factors like diabetes, hypertension and obesity. SARS-COV-2 infection may present as manifestations variety of cardiovascular manifestations including acute coronary syndrome, acute ventricular arrhythmias, heart failure, cardiomyopathy, carcinogenic shock and vascular thrombosis. Acute cardiac injury with raised cardiac troponin I levels has been found in 8 to 62% of hospitalized patients with COVID-19.^3^

Myocardial injury may reflect presence of underlying baseline comorbidities and involvement of multiple systems. Presence of myocardial injury in these patients may be due to direct injury in viral myocarditis, sepsis, acute left heart failure or myocardial supply demand mismatch with underlying stable coronary artery disease. Indirect injury due to pro inflammatory and prothrombotic state leading to thrombus formation and embolization of platelet aggregates.^3,4^ Presence of myocardial injury is indicative of significant clinical consequences. This study has been carried out to understand clinical implications of myocardial injury in COVID-19 on clinical outcome of patients.

## Material & Methods

###  Study design and data collection

A total of 3450 patients who were admitted in our tertiary cardiac care center from April 2020 to May 2021 with laboratory confirmed COVID-19 according to World Health Organizing guidelines^5^ were enrolled in the present retrospective observational cohort study. Patients aged under 18 years, without required laboratory examinations for study, patients without any clinical data, and patients with acute coronary syndrome diagnosed on ECG and as per clinical history were excluded from the study. The study was approved by institutional ethic committee IEC no. UNMICRC/Allied/2021/21.

 Data of patient’s demographics, cardiovascular risk factors, past medical history, comorbidities, laboratory parameters (routine blood test, cardiac biomarkers and inflammatory biomarkers), CT severity score (CTSS), length of hospital stay, length of ICCU admission and mortality data were obtained from standardized clinical electronic medical records. A total of 1821 patients with all the study parameters present in their electronic medical records were included in the study. All data were verified and entered into the spread sheet by the three experienced healthcare research workers.

 Patients were grouped according to survival and non-survival during hospital stay. Further the survived patients were sub grouped into two groups according to their hospital stay ≤ 10 days and > 10 days. Maximum abnormal value of TNI during the hospital course was taken for the analysis of patients with myocardial injury and patients were divided according to the level of hs-TNI into two groups TNI < 26 ng/l and TNI ≥ 26 ng/l

###  Definition 

Chronic kidney diseasewas defined as kidney damage or glomerular filtration rate (GFR < 60 mL/min/1.73m^2^) for 3 months or more, irrespective of cause. Myocardial injury was defined by cardiac biomarkers high sensitivity cardiac troponin I (hs-TNI) increased above the 26 ng/L. the cut-off value was derived by increased value of troponin I above 99^th^ percentile upper reference limit. For interpretation of CTSS score, a cut off of 20 was finalized, a score of < 20 being considered as mild and a score of ≥ 20 being considered as severe. The final score considered by the average of the scores given by two independent radiologists.

###  Statistical analysis

All statistical studies were carried out using SPSS program vs 20. Quantitative variables were expressed as the mean ± standard deviation and qualitative variables were expressed as percentage (%). A comparison of parametric values between two groups was performed using the independent sample *t* test. Categorical variables were compared using the chi-square test. Logistic regression was used to predict the in-hospital mortality. Mann–Whitney U test was performed for percentile analysis. Logistic regression analysis was performed to analyze the risk factors for in-hospital mortality. Cox regression model was used to analyze the hazard ratio for mortality. ROC curve analysis was performed to evaluate the prognostic threshold value of hs-TNI and area under curve was also calculated. A nominal significance was taken as a two tailed *p *value < 0.05.

## Results

The demographic details, basic comorbidities and radiological characteristic are shown in Table 1. Age, CT severity score, hypertension, diabetes, prior cardiovascular diseases and chronic kidney disease were significantly higher in patients with > 10 days hospital stay and non-survivors compared to patients with ≤ 10 days hospital stay. Laboratory findings like cardiac biomarkers troponin I, CPKMB and other inflammatory biomarkers interleukin 6, ferritin, D-dimer, CRP, neutrophil-lymphocyte ratio, WBC and procalcitonin were significantly higher in non-survivor patients and in patients with hospitalization stay > 10 days in compared to patients with ≤ 10 hospitalization stay.

**Table 1:** Comparison of baseline and laboratory examinations between two groups

**Variables **	**Survivors (N=1528)**		**Non-survivors** ** (N=293)**	***P*** ** value**
	**Hospital stay≤10 days (N=1299)**	**Hospital stay>10 days (N=229)**		
Female	449 (34.56%)	85(37.11%)	96 (32.76%)	0.5727
Male	850 (65.43%)	144 (63.16%)	1 97 (67.23%)	
Age	54.38 ± 14.33	57.29 ± 13.01	62.29 ± 12.41	0.0001^*^
CT severity score	17.29 ± 8.08	24.73 ± 9.99	29.39 ± 8.78	0.0001^*^
Hypertension	132 (10.16%)	35 (11.70%)	55 (18.77%)	< 0.0001^*^
Diabetes	91 (7.01%)	32 (10.70%)	36 (12.29%)	0.01^*^
Prior cardiovascular diseases (CVD)	40 (3.08%)	63 (27.51%)	113 (38.57%)	< 0.0001^*^
Chronic kidney disease (CKD)	35 (2.69%)	18 (6.02%)	20 (6.83.%)	< 0.0001^*^
Random blood sugar (RBS)	178.73 ± 119.20	174.24 ± 100.28	191.47 ± 115.26	0.07
Troponin I ≥ 26 ng/l	213 (16.40%)	176(76.85%)	239 (81.57%)	< 0.0001^*^
Creatine phosphokinase-MB (CPK-MB)	51.83 ± 69.72	78.56 ± 96.65	88.26 ± 99.49	0.0001^*^
Interleukin 6	29.18 ± 113.22	58.85 ± 153.05	323.20 ± 793.01	0.0001^*^
Ferritin	434.48 ± 476.94	738.53 ± 759.05	1247.72 ± 646.91	0.0001^*^
D-Dimer	875.28 ± 1422.41	2350.47 ± 3185.04	4209.37 ± 4005.68	0.0001^*^
C-reactive protein (CRP)	40.30 ± 52.58	40.47 ± 59.38	107.93 ± 89.62	0.0001^*^
Neutrophil-Lymphocyte ratio (NLR)	5.21 ± 5.48	10.44 ± 5.06	12.38 ± 5.23	0.0001^*^
Procalcitonin (ng/ml)	0.31 ± 0.79	2.42 ± 8.06	2.81 ± 6.18	< 0.0001^*^

^*^ *P* < 0.05 statistically significant.

Table 2 represents the comparison of clinical parameters in the different values of troponin I. Patients with group 2 (Troponin I ≥ 26 ng/l) were more likely to have higher age, hypertension, diabetes, prior cardiovascular diseases, CKD, average hospital stay, ICCU admission and length of ICCU stay, severe CT severity score, mortality, higher requirement of mechanical ventilation and higher value of procalcitonin.

**Table 2:** Distribution of clinical data according to myocardial injury

**Variables**	**Group-1 Troponin I (<26ng/l) (N=1192)**	**Group-2 Troponin I (≥26 ng/l) (N=628)**	***P*** ** value**
Age	54.48 ± 14.02	61.50 ± 13.27	< 0.0001^*^
Female	423 (35.5%)	201 (31.9%)	0.1387
Male	769 (64.5%)	429 (68.1%)	
Hypertension	119 (9.98%)	93 (14.81%)	0.003^*^
Diabetes	87 (7.30%)	72 (11.46%)	0.004^*^
Prior cardiovascular diseases	21 (1.76%)	29 (4.62%)	0.001^*^
CKD	33 (2.77%)	37(5.89%)	0.001^*^
Average hospital stay (days)	7.47 ± 6.01	9.49 ± 9.41	< 0.0001^*^
Requirement of ICCU admission in number of patients	239 (20.05%)	397 (63.22%)	< 0.0001^*^
Length of ICCU stay admission (days)	5.91 ± 2.48	8.09 ± 3.48	< 0.0001^*^
CT severity score	14.56 ± 5.88	20.86 ± 9.88	< 0.0001^*^
Mortality	54 (4.28%)	239 (38.06%)	< 0.0001^*^
Requirement of mechanical ventilation	09 (4.81%)	148 (26.10%)	< 0.0001^*^
Procalcitonin (ng/ml)	0.89 ± 4.26	2.37 ± 5.63	< 0.0001^*^

^*^ *P* < 0.05 statistically significant.

The percentile limits from 5^th^ to 95^th^ for various parameters like age, hospital stay, HRCT score and troponin I in both survival and non-survival groups, tabulated in Table 3 shows higher percentile values of all the variables in non-survival group as compared to survival group.

**Table 3:** Percentile value of different clinical parameters in population

**Groups **	**Variables**	**5th Percentile**	**25th Percentile**	**50th Percentile**	**75th Percentile**	**95th Percentile**	**97th Percentile**
Survivals	Age	30.00	45.00	55.00	64.00	79.00	82.00
Non-Survivals		40.95	54.00	64.00	71.00	81.05	83.00
Survivals	Hospital stay	3.00	4.00	6.00	8.00	17.00	24.00
Non-Survivals		1.00	3.00	7.00	14.00	28.00	32.43
Survivals	HRCT Score	3.00	13.00	18.00	24.00	35.00	36.00
Non-Survivals		9.65	24.75	31.00	36.00	40.00	40.00
Survivals	Troponin I	2.00	2.00	5.00	15.00	198.25	280.91
Non-Survivals		2.00	36.50	133.00	344.00	12360.65	20992.24

*P* < 0.05 statistically significant.

For the mortality data, the hazard ratio of, Severe CT score, troponin I ≥ 26 ng/l and NLR were found statistically significant (Table 4).

**Table 4:** Hazard ratio for mortality

**Variables **	**Hazard ratio (95% CI)**	***P*** ** value**
Age	1.03 (1.02-1.04)	< 0.0001^*^
Male	1.07 (0.89-1.28)	0.479^*^
Diabetes	2.66 (1.65-4.29)	< 0.0001^*^
Interleukin 6	1.001 (1.001-1.002)	< 0.0001^*^
Ferritin	1.001 (1-1.001)	0.017^*^
C-reactive protein (CRP)	1.006 (1.005-1.006)	< 0.0001^*^
Severe CTSS score	2.81 (1.74-4.52)	< 0.0001^*^
Troponin ≥ 26ng/L	4.68 (3.81-5.76)	< 0.0001^*^
Neutrophil-lymphocyte ration (NLR)	1.11 (1.09-1.12)	< 0.0001^*^

^*^ *P* < 0.05 statistically significant.

Table 5 represents the multivariate analysis for the prediction of myocardial injury. Odds of severe CTSS score, interleukin 6, higher hospital stay, ferritin, prior CVD history and CRP were significantly higher in patients with myocardial injury.

**Table 5:** Multivariate logistic regression for predictor of myocardial injury

**Variables**	**Odds ratio**	**95% CI**	***P*** ** value**
Severe CTSS score	1.95	1.18-3.23	< 0.0001^*^
Interleukin6	1.002	1.0-1.003	0.004^*^
Hospital stay	1.05	1.02-1.07	< 0.0001^*^
Ferritin	1.001	1.00-1.002	0.01^*^
C-reactive protein (CRP)	1.004	1.0-1.008	0.04^*^
Prior CVD history	1.65	1-2.73	0.05

^*^ *P* < 0.05 statistically significant.

## Discussion

Hs-TNI may act as a surrogate marker of cardiac damage and the level of troponin I are directly and proportionately associated with the degree of cardiac damage. Mortality rate of hospitalized patients was 16.09% in our study, which is comparable to other studies. ^6^ In our study, 34.5% patients had myocardial injury; these patients were found to have higher mortality (38.06%) as compared to patients without cardiac injury (4.28%). In our study, 81.57% of patients had myocardial injury in non-survivor group as compared to 25.46% of patients in survivor group. In the current study patients with group of raised Hs-TNI were found to have increased age, higher prevalence of underlying comorbidities, higher average hospital and ICCU stay, increased requirement of ICCU admission and mechanical ventilation which matches with the results of study reported by Weber et al.^6^

Reported prevalence of acute myocardial injury varies from 17 to 76%. In a retrospective study of 187 COVID 19 patients done by Guo et al,^7^ reported 28% of incidence of myocardial injury characterized by raised troponin T levels and reported in-hospital mortality of 60% in these patients as compared to 9% in patients with normal troponin I levels. A study done by Shi et al^8^ which included 671 hospitalized patients showed mortality rate of 9% in their study and they found that myocardial injury was present in 76% of patients who didn’t survive as compared to 10% of patients who survived. In study done by Zhou et al,^9^ they observed 17% incidence of cardiac injury and that all except one of these patients died. Similarly, a study done by Shi et al^10^ reported 20 % incidence of myocardial injury and these patients had much higher mortality rate (51%). Sharma et al^11^ reported 7.3% mortality in their study in patients with COVID-19, the mortality was twofold higher in patients with associated cardiovascular risk factors compared to COVID-19 patients without associated cardiac risk factors. These huge differences in the incidence and mortality of patients with acute cardiac injury could be related to differences in ethnicities of study populations, severity of disease, underlying baseline characteristics and comorbidities and may also be related to study period.

SARS-CoV-2 infection can cause myocardial injury via various mechanisms. High cardiac expression of Angiotensin 1 - converting enzyme-2, which is the target for SARS -COV-2 ‘s spike protein may be the reason for direct myocardial injury.^4^ SARS-CoV-2infection causes down regulation of expression of ACE2 proteins which are considered to have a protective effect on cardiac myocytes. Some histopathological studies suggest direct myocardial involvement by virus, while others suggest indirect effect of virus on myocardium secondary to hypoxia and systemic inflammation.^12^ We need further exploration and well-designed studies to investigate impact of acute cardiac injury on long term clinical outcomes as well as to investigate association between biochemical markers of cardiac injury and cardiac structure and function by using cardiac diagnostic imaging.

 Limitations of this study include retrospective study design and small sample size from the single center. Diagnostic evaluation of all patients with cardiac imaging and further bio marker analysis in patients with raised Hs-TNI was not done in our study. These additional data could help us in understanding and establishing association between elevated Hs-TNI and myocardial structure and function in this subset of patients.

## Conclusion

 Our study showed that mortality rate amongst the patients with acute cardiac injury was higher than the patients without acute myocardial injury. Patients with acute myocardial injury were found to have longer ICCU and hospital stay; higher in-hospital infection rate and higher need for mechanical ventilation. Presence of acute cardiac injury in patients with COVID 19 infection may indicate poor in-hospital clinical outcome.

## Acknowledgments

 We thank all our patients and clinical staff of our institute for supporting us to complete the work.

## Competing Interests

 All authors have none to declare.

## Ethical Approval

 The institutional ethics committee approved the study (UNMICRC/Allied/2021/21).

## Funding

 This work was supported by U. N. Mehta Institute of Cardiology and Research Centre itself and received no specific grant from any funding agency, commercial or not for profit sectors.
